# Molecular Epidemiology of Cryptosporidiosis in China

**DOI:** 10.3389/fmicb.2017.01701

**Published:** 2017-09-06

**Authors:** Yaoyu Feng, Lihua Xiao

**Affiliations:** ^1^College of Veterinary Medicine, South China Agricultural University Guangzhou, China; ^2^Division of Foodborne, Waterborne and Environmental Diseases, National Center for Emerging and Zoonotic Infectious Diseases, Centers for Disease Control and Prevention Atlanta, GA, United States

**Keywords:** cryptosporidiosis, *Cryptosporidium*, molecular epidemiology, zoonosis, China

## Abstract

Molecular epidemiology of cryptosporidiosis is an active research area in China. The use of genotyping and subtyping tools in prevalence studies has led to the identification of unique characteristics of *Cryptosporidium* infections in humans and animals. Human cryptosporidiosis in China is exemplified by the high diversity of *Cryptosporidium* spp. at species and subtype levels, with dominant *C. hominis* and *C. parvum* subtypes being rarely detected in other countries. Similarly, preweaned dairy calves, lambs, and goat kids are mostly infected with non-pathogenic *Cryptosporidium* species (*C. bovis* in calves and *C. xiaoi* in lambs and goat kids), with *C. parvum* starting to appear in dairy calves as a consequence of concentrated animal feeding operations. The latter *Cryptosporidium* species is dominated by IId subtypes, with IIa subtypes largely absent from the country. Unlike elsewhere, rodents in China appear to be commonly infected with *C. parvum* IId subtypes, with identical subtypes being found in these animals, calves, other livestock, and humans. In addition to cattle, pigs and chickens appear to be significant contributors to *Cryptosporidium* contamination in drinking water sources, as reflected by the frequent detection of *C. suis, C. baileyi*, and *C. meleagridis* in water samples. Chinese scientists have also made significant contributions to the development of new molecular epidemiological tools for *Cryptosporidium* spp. and improvements in our understanding of the mechanism involved in the emergence of hyper-transmissible and virulent *C. hominis* and *C. parvum* subtypes. Despite this progress, coordinated research efforts should be made to address changes in *Cryptosporidium* transmission because of rapid economic development in China and to prevent the introduction and spread of virulent and zoonotic *Cryptosporidium* species and subtypes in farm animals.

## Introduction

Cryptosporidiosis is a major cause of diarrhea and enteric disease in humans and farm animals. It has long been known as a primary cause of watery diarrhea in pre-weaned calves and lambs, responsible for significant morbidity and mortality (Holland, 1990; Naciri et al., 1999; Blanchard, 2012; Cho et al., 2013; Meganck et al., 2014). Even subclinical infection in older animals has been associated with reductions in growth, carcass weight and dressing efficiency (Jacobson et al., 2016). In zoo and pet snakes, gastric infection with *Cryptosporidium serpentis* is chronic and often fatal (Paiva et al., 2013). In birds, *C. baileyi* can cause respiratory and renal infections, resulting in high mortality (Santin, 2013). Recent human studies have implicated cryptosporidiosis as the second most important cause for moderate-to-severe diarrhea in young children in developing countries (Kotloff et al., 2013; Platts-Mills et al., 2015). Several *Cryptosporidium* species from mammals and birds, such as *C. parvum, C. meleagridis, C. canis, C. felis*, and *C. ubiquitum*, are important zoonotic pathogens, causing animal contact-associated or waterborne and foodborne cryptosporidiosis in humans (Xiao, 2010).

Because of the clinical, economic, and public health importance of *Cryptosporidium* spp., cryptosporidiosis has attracted the attention of many Chinese scientists, especially in recent years. They have made significant contributions to improved characterizations of *Cryptosporidium* spp. at species and subtype levels and understanding of their biology and transmission. These studies have led to the identification of unique characteristics of *Cryptosporidium* transmission in humans and animals in China.

## Molecular epidemiologic tools

Molecular epidemiology of cryptosporidiosis in humans and animals is the most active area of *Cryptosporidium* research within China. In these studies, genotyping and subtyping tools are widely used in the identification of infection sources and assessment of cross-species transmission of *Cryptosporidium* spp. (Xiao, 2010). Chinese scientists, in collaborations with scientists in other countries, have played a major role in developing some recent molecular epidemiologic tools for *Cryptosporidium* spp. For example, the PCR-RFLP analysis of the small subunit (SSU) rRNA gene using SspI and MboII developed by Chinese scientists (Feng et al., 2007b) has become the most popular genotyping tool for rapid differentiation of common *Cryptosporidium* species in ruminants (*C. parvum, C. bovis, C. ryanae*, and *C. andersoni* in cattle and *C. parvum, C. ubiquitum*, and *C. xiaoi* in sheep and goats). Several subtyping tools targeting the 60 kDa glycoprotein (gp60) gene have been developed for the assessment of the importance of zoonotic infections with several emerging human-pathogenic *Cryptosporidium* species such as *C. ubiquitum* and *Cryptosporidium* chipmunk genotype I (Li et al., 2014; Guo et al., 2015a). Their use in comparative analyses of human, animal, and water samples has led to the identification of host adaptation in *C. ubiquitum* and differences in the role of ruminants and rodents in human infections among geographic areas (Li et al., 2014). This was substantiated recently by multilocus sequence type (MLST) analysis of *C. ubiquitum* isolates (Tang et al., 2016). An MLST tool has also been developed for *C. andersoni* and *C. muris* (Feng et al., 2011a), and used by Chinese scientists in population genetic characterizations of gastric *Cryptosporidium* species in various areas (Wang et al., 2012; Zhao et al., 2013, 2014; Du et al., 2015; Zhao G. H. et al., 2015; Qi et al., 2016; Deng et al., 2017). It was shown that while the population structure of *C. andersoni* was clonal in Shaanxi and Heilongjiang provinces (Zhao et al., 2013, 2014), it was epidemic in Xinjiang and other regions (Wang et al., 2012; Qi et al., 2016).

More recently, procedures have been developed to facilitate whole genome sequencing (WGS) and advanced typing of human-pathogenic *Cryptosporidium* spp. (Guo et al., 2015b). The WGS and MLST tools have been used by Chinese scientists in studies of virulent and hypertransmissible *C. hominis* and *C. parvum* subtypes. Genetic recombination has been identified as a major mechanism for the emergence of these subtypes (Feng et al., 2013, 2014; Li et al., 2013; Guo et al., 2015c). WGS analysis of *Cryptosporidium* spp. has further indicated that copy number variation in two subtelomeric gene families encoding secreted MEDLE proteins and insulinase-like peptidases is involved in differences in host specificity between *C. parvum* and *C. hominis* and among host-adapted *C. parvum* subtype families (Guo et al., 2015c; Liu et al., 2016; Feng et al., 2017).

Although, molecular characterization of *Cryptosporidium* spp. is a recent development in China (Chen and Huang, 2007; Wang et al., 2008a,b,c), genotyping and subtyping tools are now widely used in the characterization of *Cryptosporidium* spp. in various animals (Karim et al., 2014; Liu et al., 2014a,b, 2015a,b; Ma et al., 2014, 2015; Qi et al., 2014, 2015a,b,d, 2016; Wang L. et al., 2014; Ye et al., 2014; Zhao et al., 2014; Du et al., 2015; Li J. et al., 2015, 2017; Liu A. et al., 2015; Li W. et al., 2015, 2017; Qi, M. Z., et al., 2015; Wang et al., 2015a,b; Zhang et al., 2015a,b; Zhao G. H. et al., 2015; Zhao Z. et al., 2015; Jian et al., 2016; Li F. et al., 2016; Li P. et al., 2016; Li Q. et al., 2016; Peng et al., 2016; Taylan-Ozkan et al., 2016; Xu et al., 2016; Yang et al., 2016; Zhang S. et al., 2016; Deng et al., 2017; Gong et al., 2017; Zou et al., 2017). The use of molecular diagnostic tools in prevalence studies has led to the identification of significant differences in the transmission of *Cryptosporidium* spp. between China and other countries.

## Characteristics and distribution of *Cryptosporidium hominis* subtypes in China

Molecular surveillance of *Cryptosporidium* spp. in untreated urban wastewater indicates that like other developing countries, *C. hominis* is the major *Cryptosporidium* species in humans in China (Feng et al., 2009; Li et al., 2012; Huang et al., 2017). The common *C. hominis* subtype families found in humans in developing countries, including Ia, Ib, Id, Ie, and If, are present in wastewater in Shanghai (Feng et al., 2009). However, several Ib subtypes that are rarely found elsewhere in the world, including IbA19G2, IbA20G2, and IbA21G2, are dominant *C. hominis* subtypes in wastewater. The occurrence of diverse *C. hominis* subtype families and divergent Ib subtypes in humans in China has been confirmed by subtype analysis of clinical specimens from pediatric patients (Table 1). Elsewhere in developing countries, humans are commonly infected with two *C. hominis* subtypes of the virulent Ib subtype family: IbA10G2 and IbA9G3, which have not been found in China (Xiao, 2010).

**Table 1:** *Cryptosporidium* species and subtypes in humans and nonhuman primates in China.

**Host**	**Location**	**Sample size**	***Cryptosporidium* positive (%)**	***C. hominis***		***C. parvum***		**Other species (No.)**	**References**
				**No. positive**	**Subtype (No.)**	**No. positive**	**Subtype (No.)**		
Human	Tianjin	–	5	5	IbA22G2 (1), IdA14 (1), IeA13G3T3 (1)	–	–	–	Peng et al., 2001
Human	Shanghai	6,284	102 (1.6%)	92	IaA14R4 (36), IaA18R4 (1), IdA19 (37), IdA14 (1), IbA19G2 (1), IgA14 (1)	–	–	*C. meleagridis* (6), *C. canis* (2), *C. felis* (2)	Feng et al., 2012
Human	Shanghai	252	34 (13.5%)	–	–	–	–	*C. andersoni* (34)	Liu H. et al., 2014
Human	Jiangsu	232	23 (9.9%)	2	–	–	–	*C. andersoni* (21)	Jiang et al., 2014
Human	Henan	1,366	11 (0.8%)	3	IbA19G2 (2), IeA12G3T3 (1)	2	IIdA19G1 (2)	*C. meleagridis* (5), *C. suis* (1)	Wang et al., 2013b
Human	Henan	–	10	9	IbA20G2 (3), IbA19G2 (2), IbA16G2 (1), IdA21 (2), IaA9R3 (1)	–	–	*C. felis* (1)	Wang et al., 2011c; Zhu et al., 2012
Human	Hubei	500	10 (2.0)	–	–	–	–	*C. meleagridis* (10)	Wang et al., 2017
Rhesus monkey	Guizhou	411	45 (10.9)	39	IdA20 (13), IeA11G3T3 (13), IaA13R8 (8), IaA13R7 (3), IaA14R7 (2), IfA16G2 (1)	5	IIcA5G3a (5)	*C. felis* (1)	Ye et al., 2012
Rhesus monkey	Shaanxi	86	6 (7.0)	–	–	1	IIdA15G1^*^ (1)	*C. andersoni* (5)	Du et al., 2015
Crab-eating macaques	Guangxi	205	1	1	IdA14 (1)	–	–	–	Ye et al., 2014
Nonhuman primates	Guangdong, Guangxi, Shanghai, Henan	266	19 (0.7)	14	IbA12G3 (7), IiA17 (1)	–	–	*C. muris* (5)	Karim et al., 2014
Squirrel monkey	Sichuan	–	1	1	IkA7G4 (1)^**^	–	–	–	Liu et al., 2015a

* *Misidentified as IIdA15G2R1 in the report (KJ917586)*.

** *This subtype was assigned to the wrong subtype family, as it differed significantly from KJ941148 (IkA15G1), which has priority over this sequence (KP314263)*.

In addition to Ib subtypes, Ia and Id appear to be common in China (Table 1), and two of the Ia and Id subtypes, IaA14R4 and IdA19, were the cause of a cryptosporidiosis outbreak in a pediatric ward in Shanghai that lasted >14 months (Feng et al., 2012). In this outbreak, only IaA14R4 was associated with the occurrence of diarrhea. As these patients were orphans from a welfare institute, poor hygiene by patients and caregivers was implicated as the cause of the outbreak. This was supported by concurrent augment in the transmission of several other enteric pathogens, including *Giardia duodenalis, Enterocytozoon bieneusi*, and *Clostridium difficile* (Wang et al., 2013a).

The occurrence these common *C. hominis* subtype families in humans in China is also supported by studies of *Cryptosporidium* spp. in captive nonhuman primates. Four of the five *C. hominis* subtype families, including Ia, Id, Ie, and If, have been found at high frequency in rhesus monkeys in a popular park in Guizhou, where humans and animals interact with each other closely (Ye et al., 2012). Divergent Ib subtypes, have been found in nonhuman primates in other studies (Table 1). Although, the source of *C. hominis* in nonhuman primates is not clear, these animals clearly can serve as potential reservoirs for this pathogen, and some of the *C. hominis* subtypes in nonhuman primates have been found in lake water frequented by these animals (Ye et al., 2012).

*C. hominis* has been recently found as a dominant *Cryptosporidium* species in horses and donkeys in China (Jian et al., 2016; Deng et al., 2017). The subtypes involved, however, mostly belong to the rare *C. hominis* subtype family Ik (Jian et al., 2016). Elsewhere in the world, Ik subtypes have been identified in horses in Algeria and Brazil (Laatamna et al., 2015; Inacio et al., 2017), indicating this subtype family is a host-adapted *C. hominis* with only limited public health significance. This was supported recently by WGS analysis of a human Ik isolate from Sweden, which showed that it has a much more divergent genome compared with common *C. hominis* subtypes (Sikora et al., 2016).

## Characteristics of *Cryptosporidium parvum* transmission in China

There are some substantial differences in the transmission of *C. parvum* between China and other countries, especially in preweaned dairy calves. Two large scale studies in Henan showed that although the distribution of *Cryptosporidium* species in postweaned dairy cattle was similar to the one observed in industrialized nations, preweaned dairy calves were mainly infected with *C. bovis* instead of *C. parvum* (Wang et al., 2011a,b). This was supported by subsequent studies in Heilongjiang, Shaanxi, Gansu, Ningxia, and Shanghai (Zhang et al., 2013a, 2015a; Qi, M. Z., et al., 2015; Cai et al., 2017), although in some other areas *C. parvum* was shown to be common in preweaned dairy calves, especially on large farms or farms experiencing diarrhea outbreaks (Cui et al., 2014; Huang et al., 2014; Qi et al., 2015c; Li F. et al., 2016). Elsewhere in the world, *C. parvum* is the dominant *Cryptosporidium* species in preweaned dairy calves (Xiao, 2010), and the common occurrence of *C. bovis* in this age group has only been reported in a few studies in Sweden and Malaysia (Silverlas et al., 2010; Muhid et al., 2011). In contrast, *C. bovis* is a common species in preweaned beef cattle, which are usually maintained in less extensive animal management systems (Ng et al., 2011; Rieux et al., 2013a,b, 2014; Bjorkman et al., 2015). The short history of concentrated animal feeding operations in China could be responsible for the low occurrence of *C. parvum* in preweaned dairy calves.

Subtyping of *C. parvum* in bovine studies identified the exclusive occurrence of IId subtypes in dairy calves in China, mostly IIdA15G1 and IIdA19G1 (Table 2). One study identified the exclusive occurrence of a few IIa subtypes in yaks in Qinghai (Mi et al., 2013), but this was not supported by data from other studies conducted in the same area (Qi et al., 2015a; Li P. et al., 2016). In contrast, preweaned dairy calves in other countries are mostly infected with *C. parvum* IIa subtypes, especially IIaA15G2R1, with IId subtypes common only in some areas in Sweden, Romania, Egypt, and Malaysia (Amer et al., 2010, 2013; Muhid et al., 2011; Silverlas et al., 2013; Bjorkman et al., 2015; Vieira et al., 2015; Ibrahim et al., 2016). The source of *C. parvum* IId subtypes in dairy cattle is not clear (Wang et al., 2014b). The two dominant subtypes in cattle, IIdA15G1 and IIdA19G1, are apparently common in various rodents in China (Table 3). Elsewhere in the world, rodents are seldom infected with *C. parvum* (Feng et al., 2007a), and when infected, mostly with IIa subtypes (Danisova et al., 2017). Cross-species transmission of IId subtypes is apparently common in China, as other grazing animals such as goats, horses, donkeys, and takins are also known to be infected with IId subtypes (Mi et al., 2014; Qi et al., 2015a; Zhao G. H. et al., 2015; Jian et al., 2016; Peng et al., 2016). The two dominant IId subtypes have been further identified in humans and nonhuman primates in China, suggesting the potential occurrence of zoonotic transmission of *C. parvum* (Wang et al., 2013b; Du et al., 2015).

**Table 2:** Common occurrence of *Cryptosporidium bovis* and dominance of *Cryptosporidium parvum* IId subtypes in preweaned dairy calves in China.

**Area**	**No. positive/No. examined (%)**	**Species (No.)**	***C. parvum* subtype (No.)**	**References**
Henan	172/801 (21.5)	*C. bovis* (65), *C. parvum* (54), *C. ryanae* (19), *C. andersoni* (12)	IIdA19G1 (67)	Wang et al., 2011b
Heilongjiang	72/151 (47.7)	*C. bovis* (34), *C. andersoni* (26), *C. ryanae* (5), *C. meleagridis* (5), *C. parvum* (2)	IIdA19G1 (1)	Zhang et al., 2013a
Ningxia	49/158 (31)	*C. parvum* (48), *C. bovis* (1)	IIdA15G1 (51)	Cui et al., 2014
Ningxia	19/186 (10.2)	*C. parvum* (15), *C. bovis* (4)	IIdA15G1 (15)	Huang et al., 2014
Ningxia and Gansu	122/877 (14.0)	*C. bovis* (62), *C. ryanae* (23), *C. andersoni* (19), *C. parvum (18)*	IIdA15G1 (18)	Zhang et al., 2015a
Xinjiang	37/237 (15.6)	*C. parvum* (22), *C. bovis* (9), *C. ryanae* (1), *C. andersoni* (2)	IIdA15G1 (11), IIdA14G1 (4),	Qi et al., 2015c
Shaanxi	46/186 (24.7)	*C. bovis* (22), *C. andersoni* (13), *C. ryanae* (11)	–	Qi, M. Z., et al., 2015
Beijing	14/404 (3.5)^*^	*C. parvum* (10), *C. andersoni* (4)	IIdA15G1 (4), IIdA19G1 (1), IIdA17G1 (1)	Li F. et al., 2016
Shanghai	303/818	*C. bovis* (199), *C. parvum* (72), *C. ryanae* (38)	IIdA19G1 (66)	Cai et al., 2017

* *Animals < 1 year*.

**Table 3:** Rodents and other animals as possible sources of *Cryptosporidium parvum* IId subtype family in dairy cattle in China.

**Animal**	**Location**	**Sample size**	**No. *Cryptosporidium* positive (%)**	**No. of *C. parvum***	**Subtype**	**References**
Golden hamster	Henan	50	16 (32.0)	4	IIdA15G1 (4)	Lv et al., 2009
Siberian hamster	Henan	51	4 (7.8)	2	IIdA15G1 (2)	
Campbell hamster	Henan	30	3 (10.0)	2	IIdA15G1 (2)	
Siberian chipmunk	Henan	20	6 (30.0)	2	IIdA15G1 (2)	
Brown rat	Henan	168	11 (6.6)	9	IIdA15G1 (9)	Zhao Z. et al., 2015
Golden takin	Shaanxi	191	15 (7.9)	2	IIdA19G1 (2)	Zhao G. H. et al., 2015
Yak	Qinghai, Gansu, Sichuan, Tibet	545	22 (4.0)	12	IIdA15G1 (3), IIdA19G1 (1), IIdA18G1 (1)	Qi et al., 2015a
Goat	Shanghai	302	33 (10.9)	11	IIdA19G1 (8), IIaA17G2R1 (1), IIaA15G2R1 (1)	Mi et al., 2014
Horse	Sichuan, Gansu, Inner Mongolia	–	5	4	IIdA19G1 (3)	Jian et al., 2016
Donkey	Henan, Shandong	–	82	18	IIdA19G1 (18)	
Rhesus monkey	Shaanxi	86	6 (7.0)	1	IIdA15G1 (1)	Du et al., 2015

## Transmission of other zoonotic *Cryptosporidium* spp. in humans in China

In addition to *C. hominis* and *C. parvum*, several other *Cryptosporidium* species that are traditionally associated with animals, such as *C. meleagridis, C. canis*, and *C. felis*, have been found in humans in China (Table 1). This is not surprising considering the frequent reports of these pathogens in humans in other developing countries (Xiao, 2010). As these pathogens were identified in children in urban areas in the absence of *C. parvum* (Feng et al., 2012; Wang et al., 2017), anthroponotic transmission of these *Cryptosporidium* species cannot be ruled out. In contrast, *C. andersoni* has been identified at high frequency in humans in two urban studies in China (Table 1). The unusual high *Cryptosporidium* infection rates, especially in adult patients (Jiang et al., 2014), and the identification of the canine-specific assemblage C as the dominant *G. duodenalis* genotype in one of the studies (Liu H. et al., 2014) are some other unusual observations in these studies.

Zoonotic infection appears to be important in cryptosporidiosis epidemiology in rural China, as indicated by the high ratio of zoonotic *Cryptosporidium* spp. (*C. parvum, C. meleagridis*, and *C. suis*) in HIV+ patients in a case-control study in Henan, which identified animal contact as a risk factor for cryptosporidiosis occurrence in the study population (Wang et al., 2013b). Subtype analysis identified the *C. parvum* as IIdA19G1, which is one of the two major *C. parvum* subtypes in calves, rodents, and other animals in China (see below).

## Transmission of other *Cryptosporidium* spp. in animals in China

The transmission of *Cryptosporidium* spp. appears to be unique in sheep and goats. Although, initial studies in Henan and Sichuan indicated a frequent occurrence of the zoonotic pathogen *C. ubiquitum* in sheep (Wang Y. et al., 2010; Shen et al., 2011), more recent studies have shown a common occurrence of the non-pathogenic *C. xiaoi* in sheep in Inner Mongolia and Qinghai (Ye et al., 2013; Li P. et al., 2016). Goats in China are also mostly infected with these two *Cryptosporidium* species, although in some areas a few *C. parvum* and *C. andersoni* infections were detected (Mi et al., 2014; Wang et al., 2014a; Peng et al., 2016). Elsewhere in the world, sheep and goats in European countries are frequently infected with the pathogenic and zoonotic species *C. parvum*, whereas those in Americas are mainly infected with *C. ubiquitum*. All three *Cryptosporidium* species appear to be common in sheep in Australia (Ye et al., 2013; Ryan et al., 2014). *Cryptosporidium xiaoi* appears to be common in sheep and goats in Africa, where like in China animal management is less intensive (Soltane et al., 2007; Mahfouz et al., 2014; Parsons et al., 2015; Hijjawi et al., 2016).

There are quite a few molecular epidemiologic studies of *Cryptosporidium* spp. in pigs, but results obtained thus far are in agreement with observations in industrialized nations (Wang R. et al., 2010; Chen et al., 2011; Yin et al., 2011, 2013; Zhang et al., 2013b; Lin et al., 2015; Zou et al., 2017). As in other countries, pigs in China are mostly infected with *C. suis* and *C. scrofarum*, with different distribution of these two between piglets and adults. The former is preferentially found in preweaned piglets whereas the latter is mostly found in postweaned pigs (Wang R. et al., 2010; Yin et al., 2013; Zhang et al., 2013b), although in one study *C. suis* was also found in postweaned and adult pigs at high frequency (Lin et al., 2015). *Cryptosporidium scrofarum* has been identified in Eurasian wild boars in China (Li W. et al., 2017). In contrast, both *C. suis* and *C. scrofarum* are common in these animals in Europe (Garcia-Presedo et al., 2013; Nemejc et al., 2013).

Molecular characterizations of *Cryptosporidium* spp. in other animals are less systematic. There are a few studies on the distribution of *Cryptosporidium* spp. in dogs and cats in China, but likely in most other areas, only *C. canis* and *C. felis* were identified in these animals, respectively (Jian et al., 2014; Li W. et al., 2015; Xu et al., 2016). As expected, these two species have been also found in wild or captive canine and feline animals such foxes, raccoon dogs, and mauls (*Felis manul*) (Li J. et al., 2015; Zhang S. et al., 2016; Zhang X. X. et al., 2016). As expected, host-adapted *Cryptosporidium* genotypes have been identified in the a few wild mammals examined, such as minks, bats, and other rodents (Wang et al., 2008b; Lv et al., 2009; Wang W. et al., 2013; Zhao Z. et al., 2015; Li Q. et al., 2016; Zhang S. et al., 2016). Similarly, a new *Cryptosporidium* genotype related to the bear genotype has been identified in giant panda (Liu et al., 2013), although *C. andersoni* appears to be common in both giant and less panda (Wang et al., 2015a,b). Interestingly, *C. ubiquitum* has been identified in pet chinchillas and hedgehogs in China (Qi et al., 2015b; Li Q. et al., 2016). All 11 *C. ubiquitum* isolates from chinchillas belonged to the XIId subtype family previously identified in the United States. Two novel subtype families of *C. ubiquitum* related to XIId have been found in wastewater and storm water in Shanghai, indicating *C. ubiquitum* could be a human pathogen in China (Huang et al., 2017).

## Sources of *Cryptosporidium* contamination in water

Because water exposure is frequently associated with human cryptosporidiosis in industrialized nations, extensive efforts have been made to understand the transport of *Cryptosporidium* oocysts in environment. The host-adapted nature of most *Cryptosporidium* spp. has led to the use of genotyping tools to assess the source and human-infective potential of *Cryptosporidium* oocysts in water (Xiao et al., 2000). Due to cost issues, these types of studies are largely restricted to industrialized nations. With the implementation of new Standards for Drinking Water Quality (GB 5749-2006) (http://www.iwa-network.org/filemanager-uploads/WQ_Compendium/Database/Selected_guidelines/016.pdf) in China in July 2012, *Cryptosporidium* spp. and *G. duodenalis* are now among six microbial parameters in the national drinking water quality standard, which has generated increased interest in monitoring for *Cryptosporidium* oocysts in drinking water and source water, as China is the major source for point and nonpoint *Cryptosporidium* emission to surface water (Hofstra et al., 2012; Hofstra and Vermeulen, 2016). Currently, the cost of *Cryptosporidium* detection per water sample in China is >10,000 RMB (>$1,500). As a result, regulatory testing of water samples for *Cryptosporidium* spp. is, in essence, restricted to large utilities and special events, and genotyping of *Cryptosporidium* spp. in water remains a research effort only in a few elite academic laboratories.

Procedures have been developed in China to genotype *Cryptosporidium* spp. in source water using oocyst concentration by flocculation instead of by expensive cartridge filtration and immunomagnetic separation, and detection of *Cryptosporidium* spp. by PCR instead of immunofluorescence microscopy (Feng et al., 2011b). Compared with the USEPA Method 1623 (USEPA, 2012), the new test cannot provide accurate quantitative data on the contamination level, but is much less expensive and can assess the source and human-infective potential of *Cryptosporidium* oocysts. This technique has also been used effectively in assessing the human-infective potential of *Cryptosporidium* spp. in treated urban wastewater (Ma et al., 2016). The recent development of three real-time PCR assays for genotyping and source tracking *Cryptosporidium* spp. in water could further promote research on *Cryptosporidium* contamination in drinking source watershed (Li N. et al., 2015).

The use of genotyping tools in the analysis of source water has led to the identification of *C. andersoni* and *C. suis* as the dominant *Cryptosporidium* species in Huangpu River, suggesting that cattle and pigs are major contributors to *Cryptosporidium* contamination for this drinking water source (Feng et al., 2011b). Similar observations have also been made in other areas in China using other approaches. In these studies, two poultry *Cryptosporidium* species, *C. baileyi* and *C. meleagridis*, also appear in source water in China at higher frequencies than in other countries (Table 4). In other countries, *C. suis, C. baileyi*, and *C. meleagridis* are rarely identified in drinking water sources.

**Table 4:** *Cryptosporidium* species in drinking source water in China.

**Area**	**Sample size**	**No. positive (%)**	***Cryptosporidium* species**	**References**
Shanghai	50	17 (34.0)^*^	*C. andersoni* (14), *C. suis* (7), *C. baileyi* (2), *C. meleagridis* (1), *C. hominis* (1)	Feng et al., 2011b
Shanghai	178	67 (37.6)^**^	*C. andersoni* (38), *C. suis* (27), *C. baileyi* (16), *C. scrofarum* (8), *C. meleagridis* (4), *C. parvum* (3), *C. hominis* (2), *C. ryanae* (1), *C. cuniculus* (1), *C. fragile* (1), rat genotype IV (1), avian genotype II (1), avian genotype III (1)	Hu et al., 2014
Chongqing	66	19 (28.8)	*C. andersoni* (9), *C. hominis* (6), *C. suis* (5), *C. bovis* (3), *C. meleagridis* (1), *C. baileyi* (1)	Xiao et al., 2013
Zhejiang	47	37 (78.7)	*C. suis* (2), avian genotype III (2*), C. scrofarum* (1), *C. ubiquitum* (1), *C. fragile* (1)	Xiao et al., 2012

* *Seven samples (41.2%) contained more than one Cryptosporidium species, including six samples containing two species and one containing three species*.

** *Including unspecified number of samples with mixed Cryptosporidium species/genotypes*.

The importance of pigs in *Cryptosporidium* contamination in source water in China was supported by results of the investigation of a pig carcass incident in March 2013, in which more than 16,000 pig carcasses that had been dumped in Jiaxing, Zhejiang Province reached Shanghai via the upper Huangpu River. Much higher *Cryptosporidium* detection rates were obtained in Huangpu River samples collected upstream of Shanghai during and shortly after the carcass incident, especially the occurrence of *C. suis* and *C. scrofarum*, another *Cryptosporidium* species in pigs (Hu et al., 2014). This was further supported by data on the occurrence of pig-adapted *E. bieneusi* genotypes in the river water samples.

## Challenges and opportunities of *Cryptosporidium* research in China

Despite recent progress in *Cryptosporidium* research in China, there remain some challenges. Most studies in this area have used established molecular diagnostic tools (SSU rRNA-based PCR-RFLP tool for species identification and gp60-based PCR-sequencing for subtyping) in prevalence studies. There is a lack of integrated usage of advanced molecular diagnostic tools and sophisticated epidemiological design in field studies of cryptosporidiosis. Studies are urgently needed to address public health issues related to the rapid economic development in China, such as changes in incidence and epidemiology of cryptosporidiosis in humans, impacts of concentrated animal feeding operations on *Cryptosporidium* transmission and environmental contamination, and potential introduction and dispersal of virulent and zoonotic *Cryptosporidium* species and subtypes from the Belt and Road countries. Efforts also could be made to substantiate some unusual molecular epidemiologic observations of cryptosporidiosis in China by research teams, such as the finding of *C. tyzzeri* and *C. serpentis* in several species of farm animals (Chen and Huang, 2007, 2012; Chen and Qiu, 2012) and the unexpectedly high occurrence of *C. andersoni* in urban human populations (Jiang et al., 2014; Liu H. et al., 2014). These observations are in stark contrast to other reports, and have important public health and regulatory implications.

For young scientists new to research, now may be the best time to conduct research on *Cryptosporidium* spp. In the past few years, the identification of cryptosporidiosis as a major cause for moderate-to-severe diarrhea in young infants in developing countries, the increasing occurrence of waterborne cryptosporidiosis in industrialized nations because of the chlorine–resistant nature of the parasite, and the lack of effective treatment and vaccines have attracted the attention of funding agencies. With new research investments and involvements of scientists from other research areas, major advances have been made in basic research on *Cryptosporidium* spp., including *in vitro* cultivation, cryopreservation, WGS, genetic manipulation, and drug development (Guo et al., 2015b; Vinayak et al., 2015; Morada et al., 2016; Hulverson et al., 2017; Manjunatha et al., 2017). They likely will promote research in pathogen biology of *Cryptosporidium* spp. and improve the depth of molecular epidemiological studies of *Cryptosporidium* spp. With these developments, Chinese *Cryptosporidium* research may be able to broaden its scope and increase its impact.

## Author contributions

YF and LX conducted data collection and analysis and prepared the report.

### Conflict of interest statement

The authors declare that the research was conducted in the absence of any commercial or financial relationships that could be construed as a potential conflict of interest. The reviewer MY and handling Editor declared their shared affiliation.
